# Management quality indicators and in-hospital mortality among acute coronary syndrome patients admitted to tertiary hospitals in Ethiopia: prospective observational study

**DOI:** 10.1186/s12873-021-00433-3

**Published:** 2021-03-31

**Authors:** Korinan Fanta, Fekede Bekele Daba, Elsah Tegene, Tsegaye Melaku, Ginenus Fekadu, Legese Chelkeba

**Affiliations:** 1 Department of Clinical Pharmacy, Institute of Health, Jimma University, P.O.Box: 378, Jimma, Oromia Ethiopia; 2 Department of Internal Medicine, Institute of Health, Jimma University, Jimma, Oromia Ethiopia; 3 Department of clinical pharmacy, Institute of Health Sciences, Wollega University, Nekemte, Oromia Ethiopia; 4 School of Pharmacy, Faculty of Medicine, The Chinese University of Hong Kong, Shatin New territory, Hong Kong; 5 Department of Pharmacology and Clinical Pharmacy, Collage of Health Science, Addis Ababa University, Addis Ababa, Ethiopia

**Keywords:** Acute coronary syndrome, Myocardial infarction, Management quality indicators, Mortality, Sub-Saharan Africa

## Abstract

**Background:**

Acute coronary syndrome (ACS) remains the leading cause of cardiovascular disease mortality and morbidity worldwide. While the management quality measures and clinical outcomes of patients with ACS have been evaluated widely in developed countries, inadequate data are available from sub-Saharan Africa countries. So, this study aimed to assess the clinical profiles, management quality indicators, and in-hospital outcomes of patients with ACS in Ethiopia.

**Methods:**

A Prospective observational study was conducted at two tertiary hospitals in Ethiopia from March 2018 to November 2018. The primary outcome of the study was in-hospital mortality. Data were analyzed using SPSS version 23.0. Multivariable cox-regression was conducted to identify predictors of time to in-hospital mortality. Variable with *p* -value < 0.05 was considered statistically significant.

**Results:**

Among 181 ACS patients enrolled, about (61%) were presented with ST-elevation myocardial infarction (STEMI). The mean age of the study participant was 55.8 ± 11.9 years and 62.4% were males. The use of guideline-directed medications within 24 h of hospitalization were sub-optimal (57%) [Dual antiplatelet (73%), statin (74%), beta-blocker (67%) and ACEI (61%)]. Only (7%) ACS patients received the percutaneous coronary intervention (PCI). Discharge aspirin and statin were high (> 90%) while other medications were sub-optimal (< 80%). The all-cause in-hospital mortality rate was 20.4% and the non-fatal MACE rate was 25%. Rural residence (AHR: 3.64, 95% CI: 1.81–7.29), symptom onset to hospital arrival > 12 h (AHR: 4.23, 95% CI: 1.28–13.81), and Cardiogenic shock (AHR: 7.20, 95% CI: 3.55–14.55) were independent predictors of time to in-hospital death among ACS patients.

**Conclusion:**

In the present study, the use of guideline-directed in-hospital medications was sub-optimal. The overall in-hospital mortality rate was unacceptably high and highlights the urgent need for national quality-improvement focusing on timely initiation of evidence-based medications, reperfusion therapy, and strategies to reduce pre-hospital delay.

**Supplementary Information:**

The online version contains supplementary material available at 10.1186/s12873-021-00433-3.

## Introduction

Ischemic heart disease (IHD) remains the leading cause of cardiovascular disease mortality and morbidity worldwide [1]. The mortality rate from IHD is slowly trending down in most high-income countries with advancements in the acute management of acute coronary syndromes (ACS) such as coronary revascularization, primary and secondary preventive measures after myocardial infarction and better treatment of IHD risk factors [2, 3]. However, IHD mortality rate in low and middle-income countries (LMICs) are sharply increasing as a result of an increase in cardiovascular risk factors and globalization [4, 5].

ACS is a forgotten domain in sub-Saharan Africa (SSA) since it was historically believed to be uncommon [6]. However, in recent years, SSA has faced a rapid increase in IHD risk factors, including hypertension, diabetes, dyslipidemia, obesity, and physical inactivity augmented by rapid urbanization and globalization [7]. On top of such demographic and epidemiologic transition, SSA countries have few interventional cardiology facilities equipped with cardiac catheterization laboratories [8, 9] Indeed, access to a pre-hospital emergency medical service and reperfusion therapy such as percutaneous coronary intervention and thrombolytic medications are very limited in this area [9, 10] As a result of these challenges, it is difficult to apply evidence-based Western guidelines on ACS management in most SSA countries.

While performance quality measures and clinical outcomes of patients with ACS have been evaluated widely in Western countries, inadequate data are available from SSA [11]. Indeed, the available fewer studies were limited to retrospective chart review with a small sample size, except the ACCESS-south Africa sub-analysis which prospectively enrolled large populations [12]. As a result, cardiovascular society in SSA advocate for the establishment of a prospective ACS register in SSA to increase awareness on the disease burden and strategies to optimize management protocol and clinical outcomes of ACS in the region [9].

Ethiopia, the second-most populous country in Africa (over 100 million people), is facing an increase in cardiovascular disease (CVD) burden [13]. According to the 2015 global burden of disease study, IHD is one of the top five leading causes of premature mortality in the country [14]. Despite the increasing burden of CVD in Ethiopia, the country suffers from a severe shortage of cardiologists (almost one digit) and has a few modern cardiac catheterization laboratories [15]. Facing these shortcomings, a little is known about the burden, management strategy, and clinical outcomes of ACS in the country. Therefore, this study aimed to assess the clinical profile, management quality indicators and clinical outcomes of patients with ACS admitted to two tertiary hospitals in Ethiopia.

## Methods

### Study design and clinical setting

A prospective observational study was conducted at two tertiary hospitals in Ethiopia including Jimma University Medical Center (JUMC) and Saint Peter’s Specialized Hospital (SPSH). JUMC serves as a referral center for the southwestern part of the country (over 15,000,000 catchment population). JUMC provides general internal medicine, cardiology, and other specialty services. SPSH is in Addis Ababa and it is one of a few public hospitals which provide cardiac catheterization laboratories in Ethiopia. The study was carried out from March 15 to November 15, 2018.

### Study population

All consecutive ACS patients admitted to JUMC and SPSH fulfilling the inclusion criteria were included. Patients were included if they fulfilled the following inclusion criteria: (I) age ≥ 18 years; (II) willing to provide written or oral informed consent; and (III) confirmed diagnosis of ACS based on the Third Universal Definition of Myocardial Infarction [16]. Patients who died before assessment, readmitted patients, change to the initial ACS diagnoses, and patients with non-type I myocardial infraction (secondary to non-ACS etiology) were excluded from the study. In this study, patients were categorized based on ACS subtype into ST-elevation myocardial infarction (STEMI) or non-ST-elevation acute coronary syndromes [NSTE-ACS: includes unstable angina (UA) or non-ST-elevation myocardial infarction (NSTEMI)] [17, 18].

### Data collection

Using a pre-tested structured questioner, two trained nurses and two medical interns collected clinical data by interviewing hospitalized patients and extracting relevant data from patient’s medical records. The data collection tool includes sociodemographic characteristics, key diagnostics investigations (electrocardiography, cardiac biomarkers, echocardiography, and angiography), management (guideline-directed medications within the first 24 h of hospital admission and at discharge), and in-hospital clinical outcomes [in-hospital mortality and non-fatal major adverse cardiovascular events (MACE)].

Guideline-directed in-hospital medications were defined as the initiation of dual antiplatelet (Aspirin + one P2Y_12_ inhibitor), beta blocker, and angiotensin converting enzyme (ACE) inhibitor within 24 h of hospitalization. Guideline-directed discharge medications were defined as receiving dual antiplatelet therapy (aspirin + one P2Y12 inhibitor), beta-blockers, statin, and ACE inhibitor at discharge. Guideline-directed medications were evaluated as quality of care indicators based on the international guideline for the management of STEMI or NSTE-ACS adopted at respective hospitals [17, 18].

### Study outcomes and validating tools

The primary outcome of the study was in-hospital mortality which was ascertained from treating physician death summary note. The secondary endpoint was new onset non-fatal MACE defined as a composite of heart failure, stroke, re-infarction, cardiogenic shock, and major bleeding during a hospital stay. Definition for various components of MACE was available in the supplementary material (Clinical Check-list). All clinical endpoints were recorded by following patients on daily basis from admission to discharge or death.

### Ethical approval and consent to participate

The study protocol was approved by the Institutional Review Board (IRB) of Jimma University, Institute of Health with a reference number of IHRPGD/193/18. Permission was obtained from responsible bodies of respective hospitals before interviewing patients and extracting data from active patient’s case records. Written and verbal informed consent was obtained from all study participants.

### Statistical analysis

The collected data were checked for completeness, clarity, and accuracy. Data was entered into Epidata version 4.2 and analyzed using statistical package for social science (SPSS) version 23 (IBM, Armonk, NY, USA). Patient characteristics, management, and clinical outcomes were summarized according to ACS subtypes. Continuous variables were presented as mean ± standard deviation or median (interquartile range), and categorical variables are presented as frequency (%). In-hospital ACS mortality rate was compared using Kaplan-Meier and log rank test. *P*-value < 0.25 was considered as a cut-off point to select variables on binary regression for multivariable Cox regression to determine independent predictors of time to ACS mortality. Two-tailed *p* -value < 0.05 was considered statistically significant.

## Results

Among 203 patients presented with ACS during the study period, 181 patients with a confirmed diagnosis of ACS were included. Of the181 patients enrolled, 111 (61.3%) patients had STEMI and 70 (38.7%) patients had NSTE-ACS.

### Sociodemographic characteristics

The mean age of the study participants was 55.8 ± 11.9 years and 62.4% were males. About three -fourths (75.7%) were urban or semi-urban residents while more than half of the patients had no formal education. Only 9.4% of the study participants were transported to the hospital by ambulance. The median (IQR) time from symptom onset to first medical contact was 26 (10–43) hours without significant difference (Table 1).

**Table 1:** Baseline characteristics of ACS patients admitted to selected tertiary hospitals in Ethiopia based on ACS subtypes

Patient Characteristics		All patients (*n* = 181)	STEMI (*n* = 111)	NSTE-ACS (*n* = 70)	*P*-value
Age (years)	Mean + SD	55.8 ± 11.9	55.2 ± 11.4	56.9 ± 12.7	0.349
Sex	Male	113 (62.4%)	70 (63.1)	43 (61.4)	0.825
	Female	68 (37.6)	41 (36.9)	27 (38.6)	
Residence	Urban/semi-urban	137 (75.7)	85 (76.6)	52 (74.3)	0.726
	Rural	44 (24.)	26 (23.4)	18 (25.7)	
Educational status	Illiterate	57 (31.5)	35 (31.5)	22 (51.4)	0.290
	Read and write	42 (23.2)	25 (22.5)	17 (24.3)	
	Primary school	29 (16.0)	14 (12.6)	15 (21.4)	
	Secondary and above	53 (29.3)	37 (33.3)	16 (22.9)	
Occupational status	Employed	43 (23.8)	24 (21.6)	19 (27.1)	0.526
	Merchant	55 (30.4)	37 (33.3)	18 (25.7)	
	Farmer/labor workers	30 (16.6)	20 (18.0)	10 (14.3)	
	Unemployed/retired	53 (29.3)	30 (27.0)	23 (32.9)	
Type of transportation	Ambulance	17 (9.4)	13 (11.7)	4 (5.7)	0.178
	Taxi/public bus	164 (90.6)	98 (88.3)	66 (94.3)	
Symptom onset to hospital arrival	median (IQR)	26 (10–43)	26 (8–48)	26.5 (12–40)	0.925
	≤12 h	59 (32.6)	40 (36.0)	19 (27.1)	0.214
	> 12 h	122 (67.4)	71 (64.0)	51 (72.9)	

*IQR* Interquartile range, *NSTE-ACS* Non-ST-elevation acute coronary syndrome, *STEMI* ST-elevation Myocardial Infarction, *SD* Standard deviation

### Key diagnostics and in-hospital management of ACS

The majority of the patients (79.6%) received an ECG within the first 24 h of hospital arrival and all patients received an ECG at some point during their hospital stay. Cardiac biomarkers were measured for all patients during hospitalization and 72.4% had elevated troponin I level. Diagnostic angiography was available at SPSH and it was performed for 43.6% of patients. Transthoracic Doppler echocardiography was done for 96.1 and 38.7% of the patients had reduced left ventricular ejection fraction (LVEF) less than 40% (Table 2).

**Table 2:** key diagnostics and evidence-based medications within the first 24 h and in-hospital outcomes based on ACS subtypes

Diagnostics and Medications		All patients *n* = 181	STEMI *n* = 111	NSTE-ACS *n* = 70	*P* value
Key diagnostics	ECG within 12 h	154 (79.6)	90 (81.1)	54 (77.1)	0.522
	Positive cardiac biomarker	161 (89.0)	111 (100.0)	50 (71.4)	< 0.001*
	Diagnostic Angiography	79 (43.6)	52 (46.8)	27 (38.6)	0.274
	Echocardiography	174 (96.1)	107 (96.4)	64 (95.7)	0.817
	LVEF <40%	70 (38.7)	42 (37.8)	28 (40.0)	0.771
In-hospital medications within 24 h	Aspirin	140 (77.3)	92 (82.8)	48 (68.6)	0.025*
	Clopidogrel	132 (72.9)	88 (79.3)	44 (62.8)	0.015*
	Dual antiplatelet	132 (72.9)	88 (79.3)	44 (62.8)	0.015*
	Beta-blocker (*n* = 147)	141 (67.2)	80 (72.7)	41 (58.6)	0.023*
	Any heparin	111 (61.3)	73 (65.8)	38 (54.3)	0.122
	Statin	135 (74.6)	90 (81.1)	45 (64.3)	0.011*
	ACEI/ARBs (*n* = 132)	111 (61.3)	73 (65.8)	38 (54.3)	0.449
	All GDMT^a^	103 (56.9)	71 (64.0)	32 (45.7)	0.016
In-hospital reperfusion therapy	Thrombolysis n (%)	0 (0)	0 (0)	0 (0)	–
	PCI n (%)	13 (7.2)	10 (9)	3 (3.4)	0.231
In-hospital events	Mortality	37 (20.4)	29 (26.1)	8 (11.4)	0.017
	Cardiogenic shock	16 (8.8)	13 (11.7)	3 (4.3)	0.043*
	Stroke	5 (2.8)	3 (2.7)	2 (2.9)	0.951
	Re-infraction	10 (5.5)	7 (6.3)	3 (4.3)	0.562
	Major bleeding	7 (3.9)	4 (3.6)	3 (4.3)	0.818
	Heart failure	18 (10.0)	13 (11.7)	5 (7.0)	0.317
	Non-fatal MACE	45 (25.0)	31 (28.0)	14 (20.0)	0.299
	Acute kidney injury	22 (12.2)	13 (11.7)	9 (12.9)	0.818
	Atrial fibrillation	13 (7.2)	7 (6.3)	6 (8.6)	0.565
	Hospital acquired infection	16 (8.8)	10 (9.0)	6 (8.6)	0.920

*ACEI* Angiotensin-converting enzyme inhibitor, *ARB* Angiotensin-receptor blockers, *ECG* Electrocardiography, *GDMT* Guideline-directed medical therapy, *LVEF* Left ventricular ejection fraction, *MACE* Major adverse cardiovascular events, *NSTE-ACS* Non-ST-elevation acute coronary syndrome, *PCI* Percutaneous coronary intervention, *STEMI* ST-elevation myocardial infarction

^a^GDMT: patients who received aspirin, clopidogrel, beta-blocker and ACEI within 24 h; *statistically significant at *p* < 0.05

Dual antiplatelet therapy (aspirin + clopidogrel) was administered for 73% of the patients during the first 24 h of hospital admission. The use of other guideline-directed medications includes statin (74.6%), beta-blockers (67.2%), any heparin (61.3), and ACEI/ARB (61.3) within the first 24 h of hospital admission. Patients presented with STEMI were more likely to receive evidence-based medication within the first 24 h of hospitalization compared to those presented with NSTE-ACS (Table 2).

None of the study participants received thrombolytic medications during hospitalization due to the unavailability of the medications. Although 43.6% of the participant undergone diagnostic cardiac catheterization, only 13 (7.2%) of the patients received percutaneous coronary interventions (PCI) during hospitalization.

### In-hospital complications of ACS

The overall all in-hospital mortality rate was 20.4% with significant difference between STEMI (26%) and NSTE-ACS (11.4%) [*p* = 0.017]. The rate of non-fatal MACE defined as cardiogenic shock, stroke, re-infarction, and major bleeding during hospitalization was 25% (STEMI 28% and NSTEMI 20%). Cardiogenic shock was statistically significant among patients presented with STEMI compared to NSTE-ACS patients (*p* = 0.043). There was no statistically significant difference in other non-fatal MACE among ACS subtypes. Other common complications recorded during hospitalization were acute kidney injury (12.2%), hospital acquired infections (8.8%) and atrial fibrillations (7.2%) (Table 2).

### Discharge medications

Focusing on guideline-directed discharge medications, we have evaluated prescriptions of key medications on discharge. Discharge dual antiplatelet therapy was 76% (97% for aspirin alone). Statin prescription at discharge was high (96%), followed by beta-blockers (83%) and ACEI/ARB (76%). Discharge prescriptions of aspirin and clopidogrel were more common among STEMI patients compared to NSTEM-ACS patients with statistical significance (*p* = 0.020 and *p* = 0.007), respectively (Table 3).

**Table 3:** Discharge medications according to ACS subtypes

Discharge medications	All patients (*n* = 144)	STEMI (*n* = 82)	NSTE-ACS (*n* = 62)	*p*-value
Aspirin	140 (97.2)	82 (100.0)	58 (93.5)	0.020
Clopidogrel	109 (75.7)	69 (84.1)	40 (64.5)	0.007
DAPT	109 (75.7)	69 (84.1)	40 (64.5)	0.007
Beta-blockers	120 (83.3)	72 (87.8)	48 (77.4)	0.098
ACEIs/ARBs	109 (75.7)	67 (81.7)	42 (67.7)	0.053
Statins	138 (95.8)	80 (97.6)	58 (93.5)	0.233

*ACEI* Angiotensin-converting enzyme inhibitor, *ARB* Angiotensin-receptor blockers, *NSTE-ACS* Non-ST-elevation acute coronary syndrome, *STEMI* ST-elevation myocardial infarction; ^a^*statistically significant at *p* < 0.05

### Predictors of time to in-hospital mortality

**Fig. 1:**
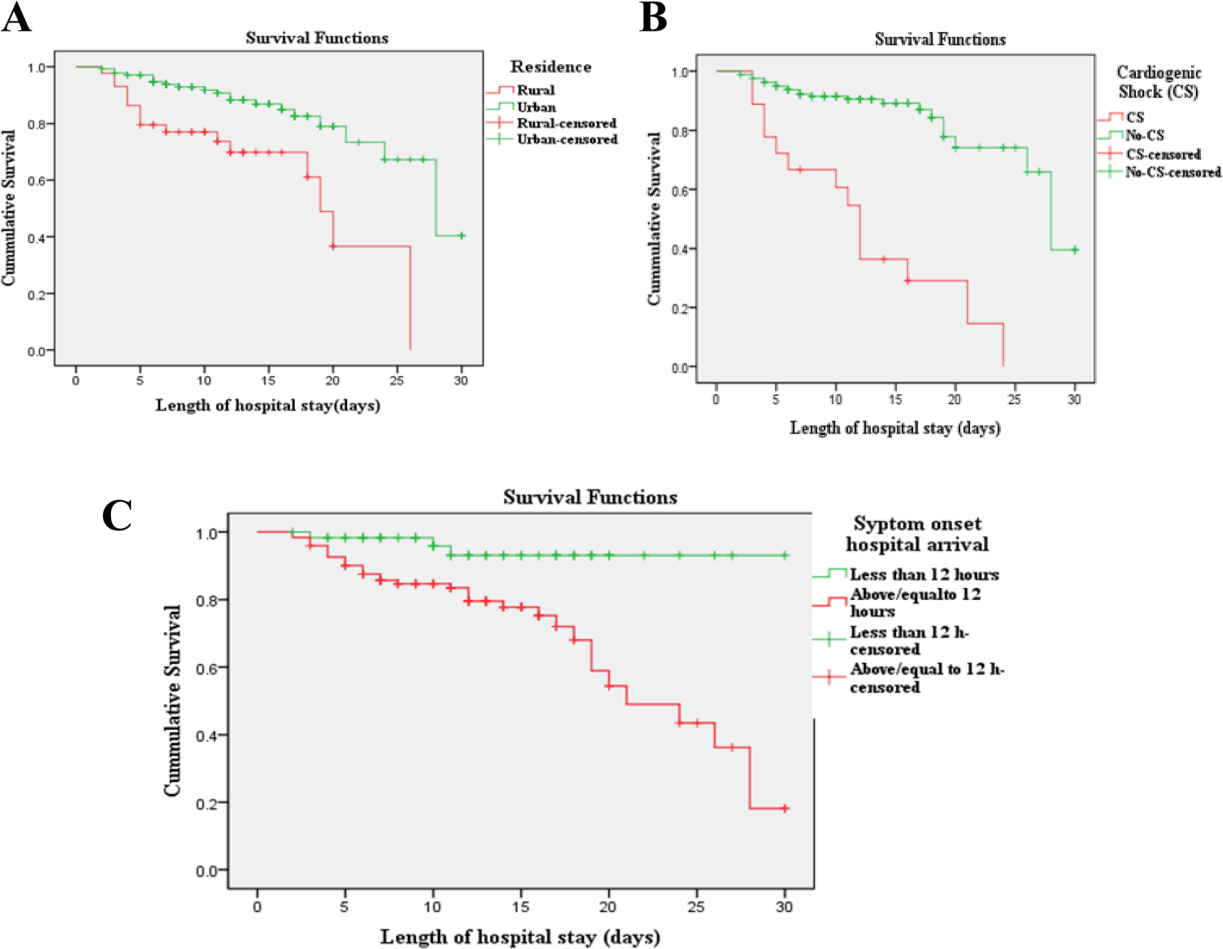
Survival probability curve derived from Log rank Kaplan Meier in-hospital mortality and residence (**a**), cardiogenic shock (**b**), and symptom onset to hospital arrival (**c**)

On multivariable Cox-regression analysis, rural residence, symptom onset to FMC > 12 h, and Cardiogenic shock complication were independent predicators of time to in-hospital death among ACS patients. The in-hospital mortality rate was 3.64 times higher in ACS patients from rural areas compared to those from urban areas [Adjusted hazard ratio (AHR): 3.64, 95% CI: 1.81–7.29). Similarly, the rate of in-hospital mortality in patients presented to the hospitals after 12 h of symptom onset was 4.23 times more than patients presented to hospital within 12 h of symptom onset (AHR: 4.23, 95% CI: 1.28–13.81). In addition, the risk of in-hospital mortality in patients who develop cardiogenic shock was 7.20 times more than those patients without cardiogenic shock (AHR: 7.20, 95% CI: 3.55–14.55). (Table 4). Survival probability curve were generated from Kaplan Meier in-hospital death for independent predictors and compared by Log rank (Fig. 1).

**Table 4:** Predictors of in-hospital mortality among ACS patients

Variables	In-hospital death (*n* = 37)	
	***Unadjusted HR (95%CI)***	***Adjusted HR (95%CI)***
Age (per year)	1.02 (0.99–1.04)	1.01 (0.98–1.04)
Residence (rural vs Urban)	3.34 (1.70–6.54)*	3.64 (1.81–7.29)*
Symptom onset to FMC > 12 h	6.08 (1.861–19.87)*	4.23 (1.28–13.81)*
STEMI vs NSTEMI (ref.)	2.03 (0.92–4.47)*	1.96 (0.84–4.56)
LVEF < 40% (≥40% ref.)	2.55 (1.3–4.98)*	0.92 (0.41–2.40)
GDMT^a^	0.43 (0.22–0.84)*	0.617 (0.31–1.23)
Atrial fibrillations	2.14 (0.88–5.21)	1.31 (0.51–3.39)
Acute kidney injury	2.69 (1.25–5.78)*	1.01 (0.39–2.61)
Cardiogenic shock	7.27 (3.66–14.44)*	7.20 (3.55–14.55)*
Re-infraction	3.47 (1.55–7.76)*	1.87 (0.78–4.46)
Hospital acquired infection	2.45 (1.11–5.38)*	1.41 (0.57–3.50)

*CI* Confidence interval, *FMC* First Medical contact, *GDMT* Guideline directed medical therapy, *HR* Hazard ratio, *LVEF* Left ventricular ejection fraction, *STEMI* ST-Elevation Myocardial infarction. ^a^GDMT (initiation of Dual antiplatelet, statin, beta-blockers, and ACEI within 24 h of hospital admission and heparin during hospital stay); *p-value < 0.005

## Discussion

Through this prospective observational study, we evaluated the process of care as quality indicators and recorded a range of in-hospital events among ACS patients admitted to two tertiary hospitals in Ethiopia. Most of our study participants were presented with STEMI in their mid-50s, with male predominance.

Prolonged pre-hospital delay (median 26 h) was recorded in our study participants which is excessively longer than the average time reported by other observational studies from low and middle income countries [19–21]. This prolonged pre-hospital delay in our study participants were mostly due to limited access to emergency medical service (only 13% used ambulance), long distance travel (especially rural residents), lack of awareness of ACS symptoms importance (more than half had no formal education), and economic reasons.

The use of guideline-directed in-hospital medications such as aspirin, clopidogrel, statin, beta-blockers, and ACEIs/ARB within the first 24 h in our study was low compared to other studies from LMICs [12, 19, 22, 23]. This might be explained by significant difference in patient level socio-economic status and lack of standard treatment protocol in our setups. There was also a significant difference in the rate of reperfusion therapy (7.2%) observed in our study compared to prior studies from SSA such as ACCESS-south Africa where 18% received thrombolytic and 53% get PCI [12]. Similarly, it was lower as compared to a study conducted at Aga Khan University, Kenya study where about 68% reperfusion rate with PCI/thrombolysis [20]. This huge discrepancy in reperfusion rate was most likely due to the unavailability of thrombolytic drugs, limited modern cardiac catheterization lab with PCI, and shortage of interventional cardiologists in our country.

The all-cause in-hospital mortality rate (20.4%) in the present study is lower compared to the study done by K. Bogale et al. [24] in Ethiopia which reported 27% and Desta DM et al. [25] in Ethiopia which reported 24.5%. However, we recorded a significantly higher in-hospital mortality rate compared to ACS registries from Kenya (17.3%) [22], South Africa (2.4% STEMI and 1.7% NSTEMI) [12], India (3.9%) [19], and Middle-East countries (7.2%) [26]. This significant discrepancy in outcome could be partly explained by differences in ACS management, particularly lack of early reperfusion therapy in our setup, prolonged prehospital delay, and high rate of non-fatal MACE observed in the present study largely contributedto this poor prognosis and higher in -hospital mortality.

The overall non-fatal MACE in the present study was 25% and without a significant difference among ACS subtypes. This finding is comparable to the ACS registry from Kenya [22] that reported a non-fatal MACE of 23%. However, the rate of no-fatal MACE recorded in our study was higher compared to observational ACS studies from India [19], Bangladesh [23], and the western European countries [27]. The higher rate of non-fatal MACE among our study participants might be due to differences in risk factors, sub-optimal guideline-directed medications, and excessive pre-hospital delay.

In the present study, rural residence, symptom onset to hospital arrival > 12 h, and Cardiogenic shock were independent predictors of time to in-hospital mortality. In this study, ACS patients from the rural areas had a three-to-four -fold increased risk of in-hospital mortality compared to those from the urban areas. Studies on ACS from developed countries also show considerable disparity when mortality is stratified by urbanization [28–30] The higher mortality rate among ACS patients from a rural area in the current study is partially explained by prolonged pre-hospital delay in rural residents and difference in socioeconomic status which could lead to a difference in treatment and related factors. Since more rural patients die before reaching hospitals, the actual death rate is likely to be greater than reported here.

Symptom onset to hospital arrival > 12 h was also an independent predictor of in-hospital mortality in the present study. This finding agrees with the previous study done by K. Bogale et al. [24] in Ethiopia which reported a significant association between delayed presentation and in-hospital mortality. Prolonged ischemic time in ACS patients has been associated with large infarct size, worse left ventricular systolic function, and more hemodynamic compromises which contribute to short-term and long-term  mortality [31, 32].

Another predictor of in-hospital mortality in the present study was a cardiogenic shock. ACS patients who developed cardiogenic shock had a 7 times higher risk of in-hospital mortality rate compared to those without cardiogenic shock. Previous studies also demonstrated that cardiogenic shock is significantly associated with early mortality among ACS patients [33–35]. A  study conducted by Desta DM et al. [25] in Ethiopia also reported high in-hospital mortality among ACS complicated with cardiogenic shock. Lack of reperfusion therapy and inotropic or vasopressors agent such as dobutamine and norepinephrine which are recommended by contemporary treatment guideline [36] as first-line  therapy for ACS complicated with cardiogenic shock also contributed to the increased mortality rate in the current study.

### Key area for quality improvement

Through this observational study, we have identified areas that need improvement to optimize the process of care and outcome among ACS patients. We observed a prolonged delay between symptom onset and presentation to hospitals for emergency care. This finding highlights the importance of creating awareness about ACS symptoms for general populations and community health workers since the prehospital delay was an independent predictor of in-hospital death in the present study. All-cause in-hospital mortality and non-fatal MACE rates in the present study were unacceptably high, stressing the importance of guideline-directed medical therapy and early reperfusion therapy. The use of guideline-directed medications such as dual antiplatelet, bet-blockers, and ACEIs should be prioritized during the acute phase (the first 24 h) since it has an impact on patient prognosis. In addition, increasing the number of interventional cardiologist and PCI cable center in available tertiary care hospitals as well as linking them with general hospitals and emergency medical service might improve outcomes of patients with ACS.

### Strength and limitations of the study

As a strength, the study was prospectively described relevant management protocol, in-hospital outcomes and independent predictors of in-hospital mortality among ACS patients. This study highlighted the process of care as quality indicators and identified area needs improvement to reduce mortality rate and complications among ACS patients in Ethiopia. However, the study has several limitations. First, this study was conducted on a small sample size (181 participants) due to erratic patient follow to selected hospitals. The calculated sample size was 190 based on the study conducted by A. Giday et al. in Ethiopia which reported an in-hospital mortality rate of 14.4% [37]. Second, the data represent patients survived the acute phase because ACS patients who died prehospital and shortly after hospital arrival were excluded. Third, we did not capture a key aspect of quality measures such as door-to-balloon/needle since only a minority of ACS patients received reperfusion therapy. Finally, we did not record economic cost, patient quality of life and post discharge medication adherence and outcomes which are also important performance measures.

## Conclusions

In the present study, the use of guideline-directed in-hospital medications was sub-optimal, and the rate of reperfusion therapy was very low (almost negligible). The rate of guideline-directed medications upon discharge was comparable to the practice of other countries and prior ACS registries report. The overall in-hospital mortality and non-fatal MACE rates were unacceptably high and highlight the urgent need for national quality-improvement focusing on early initiation of evidence-based medications, reperfusion therapy, and emergency medicals service as well as methods to enhance transportation to the hospitals.

## Supplementary Information

**Additional file 1.**

## Data Availability

The dataset that was used to support the finding of this study will be made available from the corresponding author upon reasonable request.

## Abbreviations

ACEI: Angiotensin Converting Enzyme Inhibitors
ACS: Acute Coronary Syndromes
ARB: Angiotensin Receptor Blockers
CVD: Cardiovascular Diseases
DAPT: Dual Antiplatelet Therapy
IHD: Ischemic Heart Disease
JUMC: Jimma University Medical Center
MACE: Major Adverse cardiovascular Events
NSTE-ACS: No-ST-elevations Acute Coronary Syndromes
SPSH: Saint Peter Specialized Hospital
SSA: Sub-Saharan Africa
STEMI: ST-elevations Myocardial infarction
