# Supplementary material for: Management quality indicators and in-hospital mortality among acute coronary syndrome patients admitted to tertiary hospitals in Ethiopia: prospective observational study
Source: BMC Emerg Med. 2021 Mar 31;21:41. doi: 10.1186/s12873-021-00433-3 (PMC8010978; doi:10.1186/s12873-021-00433-3)
Supplement: Supplementary file 1 — Additional file 1. [file 12873_2021_433_MOESM1_ESM.docx]

**Operational definitions**

- Non-fatal MACE was defined as composite end point of in hospital stroke, re-infraction, cardiogenic shock, heart failure and major bleeding.
- Stroke was considered when treating physician made the diagnosis based on the finding of brain computed tomography.
- Diagnosis of a re-infarction was done when recurrent myocardial infarction confirmed by ECG changes or elevation of cardiac markers (re-elevation of the CK-MB to above the ULN and increased by at least 50% over the previous value) occurred in-hospitalized acute myocardial infarction patients.
- Cardiogenic shock was confirmed when inotropes (dopamine in our case) required to achieve a blood pressure ≥90 mmHg and signs of impaired organ perfusion with at least one of the following: altered mental status, cold, clammy skin, or oliguria.
- Major bleeding was defined as the occurrence of clinically overt/apparent bleeding associated with drop in hemoglobin >5 g/dL or Intracranial or intraocular hemorrhage
- Acute kidney injury (AKI) incident was recorded when serum creatinine increased by ≥0.3 mg/dl or increase to more than or equal to 1.5 times from baseline within any 48 hours during hospital days.
- The criteria used to define incident AF included: No documentation of history of AF and either a) new-onset AF documented in any clinical note during the index hospitalization or b) AF deemed present by an interpreting cardiologist on any 12-lead ECG obtained during the index hospitalization
- Hospital acquired infection was documented when occurrence of infection diagnosed after 48 h of hospitalization in a patient otherwise not having symptomatic or incubating infection on hospital admission.
